# Traumatic dental injuries over an 8-year period at a German dental center: a retrospective overview and cross-sectional analysis

**DOI:** 10.1186/s40729-023-00506-x

**Published:** 2023-11-01

**Authors:** Puria Parvini, Yanislava Lermen, Robert Sader, Frank Schwarz, Karina Obreja

**Affiliations:** 1 Department of Oral Surgery and Implantology, Johann Wolfgang Goethe-University, ZZMK Carolinum, Theodor-Stern-Kai 07, H.29, 60590 Frankfurt, Germany; 2 Clinic for Cranio-Maxillofacial and Facial Plastic Surgery, Johann Wolfgang Goethe-University, Frankfurt, Germany

**Keywords:** Traumatic dental injury, Periodontal trauma, Hard-tissue trauma

## Abstract

**Background/aim:**

The aim of this study was to analyze a population of patients who had suffered from traumatic dental injuries (TDIs) by using different patient-, trauma- and treatment-related parameters.

**Material and methods:**

All dental records of patients ≥ 3 years old who had presented at the dental emergency service between Jan 1, 2009 and Dec 31, 2016 for the treatment of dental trauma were analyzed. A total of 2758 patients were invited for a recall examination at the Department for Dental Surgery and Implantology, ZZMK Carolinum, Goethe University Frankfurt, Germany; of these, 269 patients attended their recall appointments.

**Results:**

The enrolled patient population consisted of 1718 males and 1040 females, with a mean age of 19.63 years (median 12.00 ± 17.354 years). A total of 4909 injured teeth were assessed, with a mean of 1.78 injured teeth per patient (median 2.00 ± 1.279). Males were found to be more frequently affected by TDIs compared to females (1.65:1). The majority of these injuries occurred in the first two decades of life (66.1%; *n* = 1824). The majority of the patients presented for initial treatment within 24 h of their accident (95.7%). The most frequent TDIs were isolated luxation injuries 49.4% (*n* = 2426) and isolated crown fractures 30% (*n* = 1472). Combination injuries were diagnosed in 20.6% of the cases (*n* = 1011).

**Conclusions:**

Based on the findings of the present analysis, it can be concluded that males were more frequently affected by TDIs than females. Most patients had suffered from TDI before they had turned 10 years of age. Overall, the enamel–dentin fracture was found to be the most frequent injury, followed by concussions and lateral luxations.

**Graphical Abstract:**

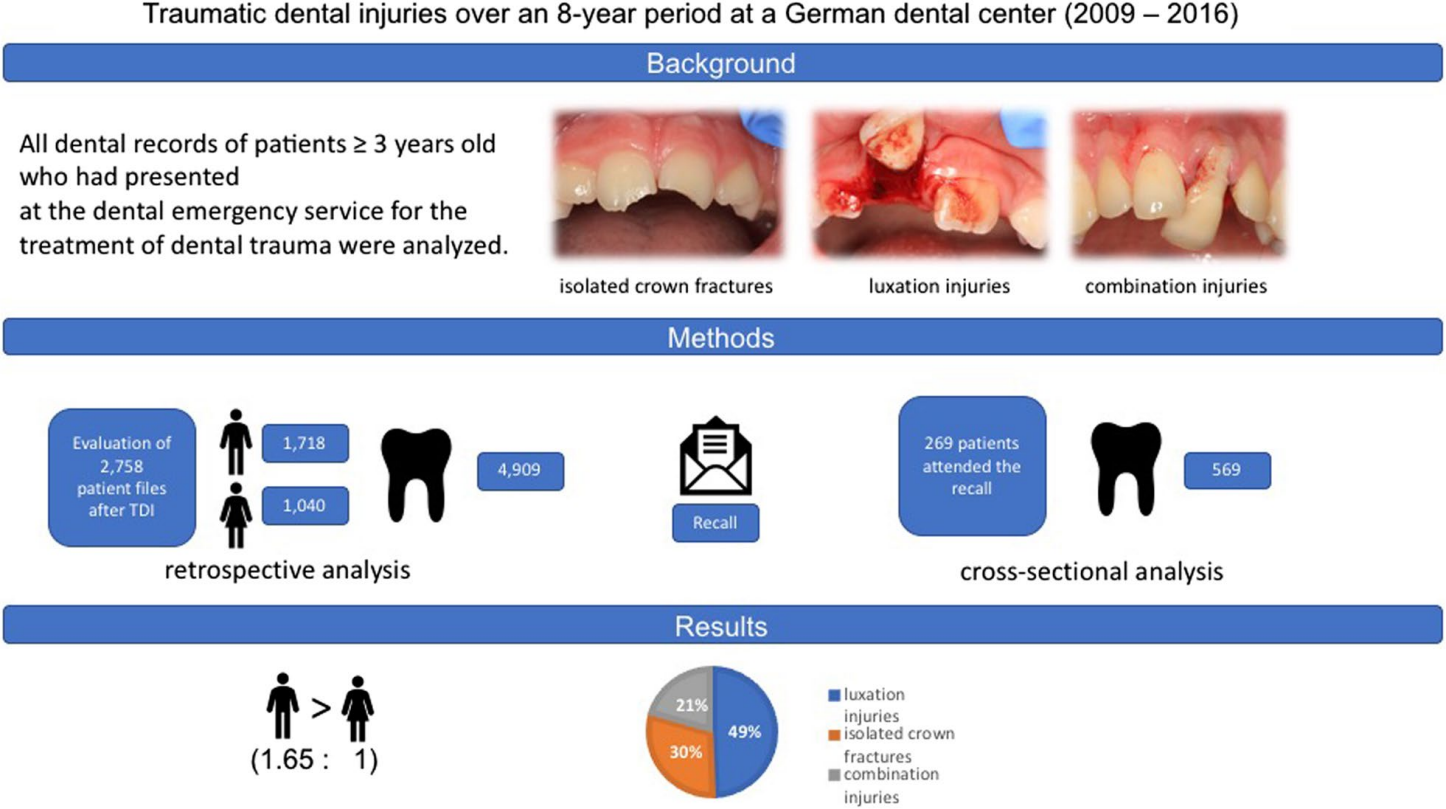

## Introduction

Traumatic dental injuries (TDI) are acute, mechanical injuries to the teeth and adjacent structures; TDIs account for 5% of all treatments carried out on permanent teeth [1]. With an estimated incidence of about 1 in 100 per year, and prevalences of 14.8% and 23.8% among the permanent and deciduous teeth, respectively, 15.2% of the current world population have, at one point in their lives, been affected by dental trauma [2, 3]. Gender and oral factors such as dental overjet with protrusion [4] and incomplete lip coverage influence [5] the prevalence of dental trauma. The growing popularity of certain sports also plays a role [6, 7] and, thus, nowadays, more than half of children and adolescents experience a dental injury before the age of 18 years. [8] The worst-case outcome of a TDI is permanent tooth loss, with all its substantial functional and psychological implications [8, 9]. Such a loss of teeth can pose particular challenges in children and adolescents where ongoing growth makes it more difficult to achieve esthetically and functionally acceptable outcomes.

When deciduous teeth are affected by traumatic injury, the benefits of preservation must be weighed against the risks to the permanent dentition. Consideration should be given to the type and extent of the damage and the progress of tooth germ development, as well as the patient's age given that there is a higher risk of permanent injury in younger children. A significant role is also attributable to the forcefulness and direction of the traumatic impact, with intrusion of the deciduous teeth arguably being the most violent example [10].

Before focusing on the teeth, the dental trauma patients need to be examined comprehensively to rule out any systemic effects of the accident such as brain injury, hemorrhage, or the fracture of bones [11]. A neurological examination of the major facial nerves is required and the mandible should be checked for mobility to exclude the possibility of jaw fracture. It is technically advisable to appraise and record on a trauma documentation form the exact course of the accident and the patient's medical and dental history.

Records from a standardized documentation form of this type, used by the dental emergency service at the authors' university center, provided the basis for designing a retrospective study of all patients who had presented with dental injuries over an 8-year period. Against a background of considerable evidence in the literature on the nature and demographic aspects of such injuries, but with less being available on their long-term implications and sequelae, it was decided to complement the retrospective analysis by inviting all patients to attend a follow-up examination for an additional cross-sectional analysis.

The aim of this study was to analyze a population of patients who had suffered from traumatic dental injuries (TDIs) by using different patient-, trauma- and treatment-related parameters.

## Materials and methods

This study was conducted by collecting the medical records of 2,758 patients ≥ 3 years of age who had been admitted to the Department for Dental Surgery and Implantology, ZZMK Carolinum, Goethe University Frankfurt, Germany; in the period of January 01, 2009 to December 31, 2016. The data of all patients were collected and analyzed by two dentists (JL and KF).

The study protocol was in accordance with the Helsinki Declaration of 1975 (revised in August 2018) and approved by the Ethics Committee Goethe University Frankfurt, Germany University.

Subsequently, all 2758 patients were invited for a follow-up visit. Written informed consent was required from each patient, or his or her legal representative, based on the comprehensive information provided about the nature, scope, benefits and risks of the study.

Demographic data (gender, date of birth) were collected from the medical records and the different types of TDI, as well as the epidemiological variables, were obtained from the standardized emergency documentation forms used at the center.

The information collected for each patient included their medical/dental history, the nature of the accident, photographs, the findings obtained in extraoral and intraoral examinations, as well as injuries not within the scope of the oral and maxillofacial regions.

The following study variables were assessed: (1) the patient's age, (2) gender, (3) oral hygiene status, (4) tooth development status, (5) previous dental injuries, (6) number of (permanent or deciduous) teeth injured, (7) number of distinct injuries, (8) general signs and symptoms, (9) accident data (time, place, reason, course of events), (10) primary care received, (11) time passed from the accident to the dental examination, (12) tetanus vaccination status, (13) diagnosis including soft tissue and bone injuries, (14) mouth opening (mm) and/ or occlusal problems, (15) treatment protocol (conservative, endodontic, or surgical including extraction), and (16) radiographic findings (tooth root and/or bone fracture).

For avulsed teeth, the details were evaluated on (1) reimplantation (yes/no), (2) extraoral time (min) and (3) the storage medium.

Dento-alveolar injuries were classified according to the classification proposed by Andreasen (1994). In addition, an injury to the periodontal tissues with a simultaneous hard dental tissue injury to the same tooth was referred to as a combination injury. The classification of the traumatic injuries was followed according to Andreasen and Andreasen (1994) (Table 1).

**Table 1:** Classification of the traumatic injuries according to Andreasen and Andreasen (1994)

*I. Injuries to the hard dental tissues and pulp*
Enamel fracture
Enamel–dentine fracture
Complicated crown fracture
*II. Injuries to the hard dental tissue, pulp and alveolar process*
Crown–root fracture
Root fracture
Alveolar fracture
*III. Injuries to the periodontal tissues*
Concussion
Subluxation
Luxation injuries
Lateral luxation
Intrusion
Extrusion
Avulsions

### Cross-sectional analysis

The patients who attended the follow-up examination (*n* = 269) provided the relevant information required to compile their comprehensive histories, detailing any initial and subsequent events and treatment steps. The series of radiographs and photographs on file helped to identify the sequelae of the original injuries and their treatment. All previously injured teeth that survived were tested using a Periotest device (Medizintechnik Gulden, Moldautal, Germany) and percussion for their mobility or ankylosis. A positive value is reflected for loose teeth. Conversely, a negative value is registered with ankylosed teeth.

All sites with tooth loss were clinically examined with respect to the sequence of events following the injury, including any effects on bone quantity and jaw growth, outcomes of the treatment provided by the tooth- or implant-supported restoration, orthodontic gap closure, or the transplantation of a deciduous or permanent tooth. Other clinical parameters included (1) tooth sensitivity, (2) probing depths on six aspects of the tooth, (3) discoloration, (4) growth inhibition, (5) patient compliance with recalls, and (6) identification of treatment requirements. Radiographs were obtained, whenever indicated, to identify periodontal or endodontic pathologies. The radiographs were evaluated for (1) apical periodontitis, (2) root fracture and/or root resorption, and (3) endodontic treatment received.

**Fig. 1:**
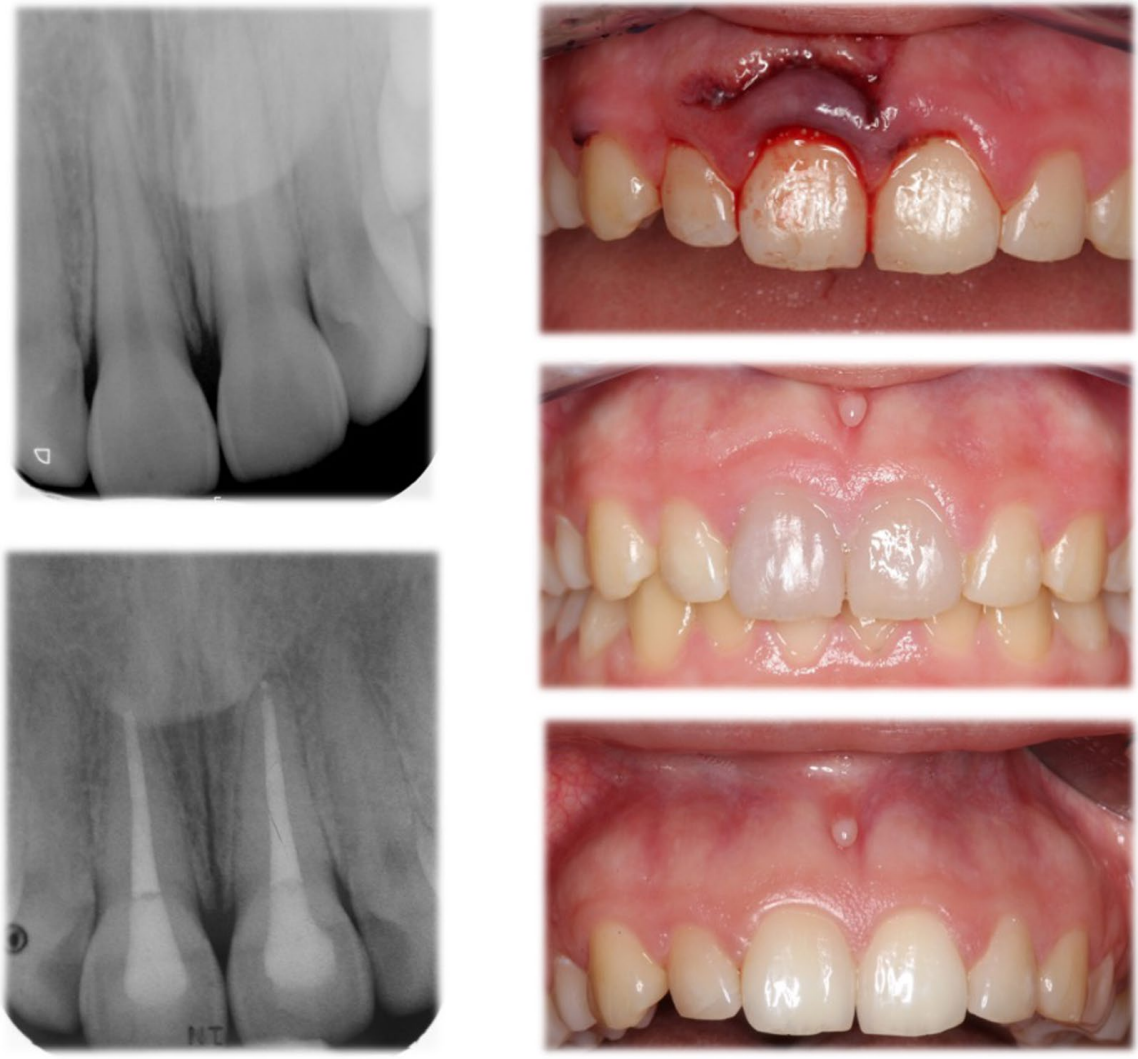
Clinical case—endodontic problem at recall

Any findings of endodontic or periodontal complications resulted in further treatment (Fig. 1). However, the patients were first provided with all necessary comprehensive information about these treatments. If any oral surgical need was identified the treatment was subsequently performed in the Department of Oral Surgery and Implantology.

### Statistical analysis

All data were entered into a spreadsheet (Excel 2019, Microsoft, Redmond, WA) and descriptive analysis was undertaken with statistical software (SPSS Statistics, version 26; IBM, Armonk, NY).

Chi-square testing was used to analyze the categorical data. All tests were implemented as two-sided significance tests with differences considered significant at *p* < 0.05. A Kolmogorov–Smirnov test was used to check the metric variable for normal distribution, however, normal distribution was not confirmed (*p* < 0.05). Hence, non-parametric tests were employed for comparisons, the primary endpoint being four major sequelae (tooth loss, hard-tissue restoration, endodontic treatment, general sequelae) of the three injury types (PDL (periodontal ligament), DHT (dental hard tissue), PDL + DHT). A secondary endpoint concerned the likelihood of implant treatment following traumatic dental injuries. Periotest values were analyzed by a Kruskal–Wallis test, while all other comparisons were analyzed by Pearson’s Chi-squared tests.

## Results

### Retrospective analysis

**Fig. 2:**
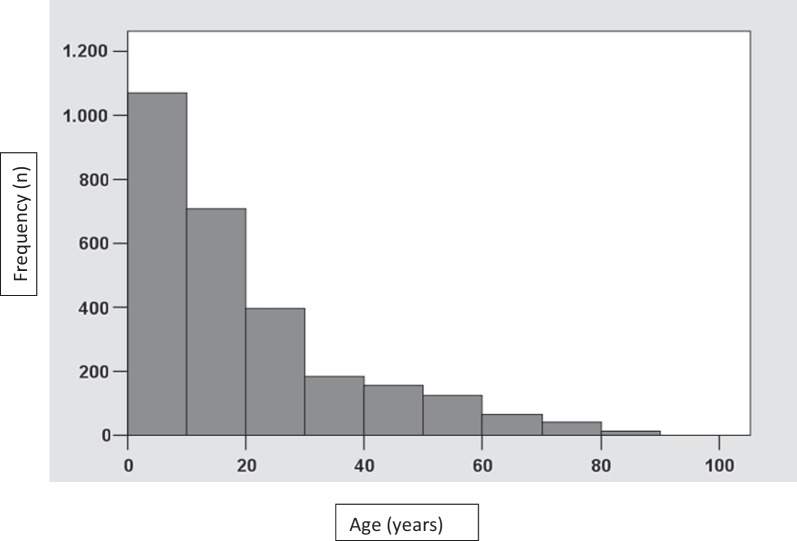
Distribution of traumatic dental injuries according to age

The study comprised 1718 male (62.3%) and 1040 female (37.7%) patients with a male-to-female ratio of 1.65:1 (Table 2). The mean age of the included patients was 19.63 ± 17.35 years (median: 12 years; range: 3–83 years). Even though children < 3 years old were excluded due to compliance issues, under-10-year-olds were still the largest group, with older decades progressively decreasing in patient numbers (Fig. 2). Under-20-year-olds (*n* = 1824) accounted for 66.1% of the sample.

**Table 2:** Overview of the retrospective total sample of patients, the cross-sectional subsample, and their traumatic dental injuries

	Retrospective		Cross-sectional	
*Patients injured*	*n*	%	*n*	%
Female	1040	37.7		
Male	1718	62.3		
Total	2758	100	269	
*Age at injury/recall*	*Mean*	*SD*	*Mean*	*SD*
Years	19.63	17.35	27.55	19.72
*Teeth injured*	*n*	*%*	*n*	*%*
Permanent teeth	4217	85.9	569	95.2
Deciduous teeth	692	14.1	29	4.8
Total	4909	100	598	4.8
PDL injuries	2426	49.4	258	45.3
DHT injuries	1472	30.0	152	26.7
PDL + DHT injuries	1011	20.6	159	27.9
Total	4909	100	569	100

*DHT* dental hard tissue, *PDL* periodontal ligament

Most of the injuries were found to have occurred in public sports or play facilities (24.8%), at home (21.9%), during bicycle falls (15.6%), on the street (11.4%) or by physical force (8.3%). Although most of the patients (*n* = 930) had presented with only one injured tooth, the extensiveness of the trauma ranged from injuries confined to the soft tissue in 330 patients to ten affected teeth in one individual (mean: 1.78 ± 1.28 injuries per patient) (Table 3).

**Table 3:** Overview of the accident environments, extensiveness of the dental trauma per patient and of the affected upper and lower permanent or deciduous teeth

Accident environments	*n*	%	Extensiveness of traumatic dental injuries		
				Patients	Sites
				*n*	*n*
Public sports/play	685	24.8	Soft tissue only	330	0
Home	604	21.9	One tooth	930	930
Bicycle	430	15.6	Two teeth	913	1826
Street	314	11.4	Three teeth	339	1017
Violence	228	8.3	Four teeth	168	672
School	164	5.9	Five teeth	37	185
Play	135	4.9	Six teeth	20	120
Work	80	2.9	Seven teeth	9	63
Fainting	68	2.5	Eight teeth	10	80
Traffic	40	1.5	Nine teeth	1	9
Epilepsy	7	0.3	Ten teeth	1	10
Alcohol	2	0.1			
Intubation	1	0.0			
Total	2758	(100)	Total	2758	4912

Maxilla						Mandible					
Permanent teeth			Deciduous teeth			Permanent teeth			Deciduous teeth		
Site	*n*	%	Site	*n*	%	Site	*n*	%	Site	*n*	%
11	1327	31.5	51	237	34.2	31	158	3.7	71	11	1.6
21	1377	32.7	61	218	31.5	41	157	3.7	81	13	1.9
12	381	9.0	52	76	11.0	32	88	2.1	72	15	2.2
22	447	10.6	62	77	11.1	42	89	2.1	82	12	1.7
13	65	1.5	53	16	2.3	33	13	0.3	73	4	0.6
23	51	1.2	63	9	1.3	43	18	0.4	83	1	0.1
14	7	0.2	54	1	0.1	34	3	0.1	74	0	0.0
24	8	0.2	64	1	0.1	44	2	0.0	84	1	0.1
15	5	0.1	55	0	0.0	35	0	0.0	75	0	0.0
25	5	0.1	65	0	0.0	45	2	0.0	85	0	0.0
16	2	0.0	–	–	–	36	3	0.1	–	–	–
26	4	0.1	–	–	–	46	1	0.0	–	–	–
17	2	0.0	–	–	–	37	2	0.0	–	–	–
27	0	0.0	–	–	–	47	0	0.0	–	–	–
Jaw ∑	3681	87.3		635	91.8		536	12.7		57	8.2
+	536	12.7		57	8.2		←	←		←	←
Total	4217	(100)		692	(100)						4909

∑ = total

The most frequently injured teeth, in both deciduous and permanent dentitions, were the upper central incisors (65.7% and 64.2%, respectively), followed by the maxillary lateral incisors (19.6% and 22.1%, respectively). Upper-jaw injuries clearly prevailed (87.9%), again based on permanent (87.3%) and deciduous (91.8%) teeth. Only 49 (46 permanent and 3 deciduous) posterior teeth were affected overall, accounting for a mere 1% of the total 4909 tooth sites.

The aforementioned mean of 1.78 ± 1.28 injuries per patient rises to 2.13 ± 1.65 if all injuries to either the periodontal ligament (PDL) or the dental hard tissue (DHT) are regarded as separate entities (Table 4). This is because 1011 (20 deciduous, 991 permanent) teeth were injured in both the PDL and DHT, accounting for 20.6% of all 4909 teeth as compared to either the DHT (1472; 30.0%) or PDL (2426; 49.4%) injuries alone. Hence, based on all the PDL injuries, the most frequent subtype of injury was concussion (31.4%), followed by lateral luxation (27.9%) and subluxation (23.5%), while for the DHT injuries, enamel–dentin injuries accounted for almost half (48.4%), followed by enamel (22.6%) and enamel–dentin–pulp injuries (11.6%).

**Table 4:** Number of injuries to the permanent and deciduous teeth, arranged according to injury types and subtypes

	Number of injuries sustained by								
Types of traumatic injury	Permanent teeth			Deciduous teeth			All injuries		
(PDL, DHT, PDL + DHT)	*n*	*n*	%	*n*	*n*	%	*n*	*n*	%
PDL (including PDL + DHT)	2780			657			3437		
Concussion		983	35.4		95	14.5		1078	31.4
Subluxation (mobility)		612	22.0		194	29.5		806	23.5
Lateral luxation		762	27.4		196	29.8		958	27.9
Extrusion		141	5.1		46	7.0		187	5.4
Intrusion		76	2.7		34	5.2		110	3.2
Avulsion		206	7.4		92	14.0		298	8.7
			100			100			100
DHT (including PDL + DHT)	2428			55			2483		
Enamel (infraction)		199	8.2		4	7.3		203	8.2
Enamel (fracture)		537	22.1		23	41.8		560	22.6
Enamel–dentin		1191	49.1		11	20.0		1202	48.4
Enamel–dentin–pulp		282	11.6		5	9.1		287	11.6
Crown–root		178	7.3		7	12.7		185	7.5
Root		41	1.7		5	9.1		46	1.9
			100			100			100
Total	5208			712			5920		

Injuries to the periodontal ligament or dental hard tissue are counted separately in this table, even if any two of them affected the same tooth site

*DHT* dental hard tissue, *PDL* periodontal ligament

Regarding the permanent teeth, the main PDL injuries were concussion (35.4%), lateral luxation (27.4%) and subluxation (22.0%), while the main DHT injuries were enamel–dentin injury (49.1%), enamel fracture (22.1%) and enamel–dentin–pulp injury (11.6%). With respect to the deciduous teeth, the main PDL injuries were lateral luxation (29.8%) and subluxation (29.5%), while the main DHT injuries were enamel fracture (41.8%) and enamel–dentin injury (22.6%). Furthermore, the main subtypes of the combined PDL + DHT injuries were enamel–dentin injury plus concussion (29.3%) Table 5 shows the injuries (PDL, DHT, PDL + DHT) to the permanent and deciduous teeth, arranged according to injury types.

**Table 5:** Injuries to the permanent and deciduous teeth, arranged according to injury types

Types of traumatic injury	Permanent teeth			Deciduous teeth			All teeth		
(PDL, DHT, PDL + DHT)	*n*	%	%	*n*	%	%	*n*	%	%
PDL injuries	1789		73.7	637		26.3	2426		100.0
% based on injury types		42.4			92.1			49.4	
DHT injuries	1437		97.6	35		2.4	1472		100.0
% based on injury types		34.1			5.1			30.0	
PDL + DHT injuries	991		98.0	20		2.0	1011		100.0
% based on injury types		23.5			2.9			20.6	
All injuries	4217		85.9	692		14.1	4909		100.0
Total %		100			100			100	

*DHT* dental hard tissue, *PDL* periodontal ligament

^*^Injuries to deciduous versus permanent teeth: *p* < 0.001 (Pearson's Chi-squared test)

A Pearson’s Chi-squared test was performed, revealing that the difference in injury types between the permanent and deciduous teeth was statistically significant (*p* < 0.001). The Pearson's Chi-squared test also revealed significant differences with regard to the number of extractions performed after the injuries (Table 6); teeth had to be removed significantly more often after PDL injuries than after DHT or PDL + DHT injuries (*p* < 0.001), while injuries to the deciduous teeth were followed by extraction significantly more often than injuries to the permanent teeth (*p* < 0.001).

**Table 6:** Teeth extracted or not extracted after sustaining traumatic injury, arranged according to injury types

Types of traumatic injury	Not extracted			Extracted			Other†			All teeth		
(PDL, DHT, PDL + DHT)	*n*	%	%	*n*	%	%	*n*	%	%	*n*	%	%
PDL injuries	2144		88.4	130		5.4	152		6.3	2426		100
% based on injury types		46.7			80.7			98.7			49.4	
DHT injuries	1444		98.1	27		1.8	1		0.1	1472		100
% based on injury types		31.4			16.8			0.6			30.0	
PDL + DHT injuries	1006		99.5	4		0.4	1		0.1	1011		100
% based on injury types		21.9			2.5			0.6			20.6	
All injuries	4594		93.6	161		3.3	154		3.1	4909		100
Total %		100			100			100			100	

*DHT* dental hard tissue, *PDL* periodontal ligament

^†^This category includes teeth that were not reimplanted (*n* = 141) or could not be found (*n* = 13) after the accident

More teeth with PDL than with DHT or PDL + DHT injuries extracted: *p* < 0.001 (Pearson's Chi-squared test)

More deciduous than permanent teeth extracted (data not shown): *p* < 0.001 (Pearson's Chi-squared test)

### Cross-sectional analysis

A total of 269 (9.8%) patients with 598 previously injured (569 permanent and 29 deciduous) teeth attended the follow-up recall (Table 1).

Most of these patients (75.1%) were not found to require additional treatment. Of the remaining patients, the diagnosed indications were for endodontic treatment in 22 (8.2%) patients, for extraction in 21 (7.8%), conservative or prosthetic treatment in 12 (4.5%), orthodontic in five (1.9%), as well as apicoectomy in four (1.5%) and the treatment of ankylosis in three (1.1%) patients. In addition, orthodontic treatment was already ongoing in 30 (11.2%) of the patients at the time of the study and had been completed in a further 44 (16.4%). This treatment was also being planned for a further 31 (11.5%) patients, however, this option was not mentioned by the remaining 164 (61.0%) patients.

**Fig. 3:**
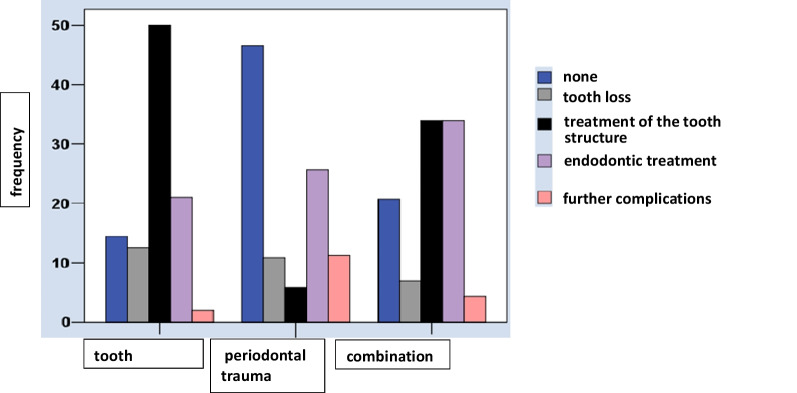
Sequelae after dental trauma

Previous injuries were recorded for the remaining 569 permanent teeth at the follow-up visits (Fig. 3). Roughly one-third of them (30.8%) had involved no events, 58 (10.2%) had been lost (almost half of these were PDL injuries) or they had been subjected to restorative (2.5%) or root-canal (26.7%) treatment, while miscellaneous, other sequelae accounted for the remainder (39; 6.85%). A Pearson’s Chi-squared test disclosed that the sequelae were significantly associated with differences in the injury type, i.e., PDL versus DHT versus PDL + DHT injuries (*p* < 0.001) (Tables 7, Table 8).

**Table 7:** Cross-sectional subsample of patients: sequelae of previous traumatic injuries to the permanent teeth

Restorative treatment provided																		
Types of traumatic injury	No sequelae			Tooth loss			Hard tissue			Endodontic			General sequelae			All teeth		
(PDL, DHT, PDL + DHT)	*n*	%	%	*n*	%	%	*n*	%	%	*n*	%	%	*n*	%	%	*n*	%	%
PDL injuries	120		46.5	28		10.9	15		5.8	66		25.6	29		11.2	258		100
% based on injury types		68.6			48.3			10.3			43.4			74.4			45.3	
DHT injuries	22		14.5	19		12.5	76		50.0	32		21.1	3		2.0	152		100
% based on injury types		12.6			32.8			52.4			21.1			7.7			26.7	
PDL + DHT injuries	33		20.8	11		6.9	54		34.0	54		34.0	7		4.4	159		100
% based on injury types		18.9			19.0			37.2			35.5			17.9			27.9	
All injuries	175		30.8	58		10.2	145		25.5	152		26.7	39		6.9	569		100
Total %		100			100			100			100			100			100	

*DHT*  dental hard tissue, *PDL*  periodontal ligament

Association between sequelae and injury types: *p* < 0.001 (Pearson's Chi-squared test)

**Table 8:** Cross-sectional subsample of patients: injury subtypes based on previous trauma to the permanent teeth in relation to both clinical and radiographic findings at follow-up and to the distribution of these subtypes within the combined PDL + DHT injurie**s**

				Status at follow-up		Endodontic treatment				Other findings (left) or	
Types of traumatic injury				Lost	Nonsensitive	Previous	Indicated	Ankylosed	Discolored	requirements (right)	
(PDL, DHT, PDL + DHT)	*n*	*n*	%	*n*	*n*	*n*	*n*	*n*	*n*	*n*	*n*
*PDL injuries*	258										
Concussion		44	17.1	0	3	2	1	3	0	1	
Subluxation (mobility)		49	19.0	1	9	6	9	2	2	7	2
Lateral luxation		94	36.4	8	34	25	6	4	14	19	
Extrusion		19	7.4	3	8	5	1	1	5	3	
Intrusion		17	6.6	1	7	3	3	0	2	14	1
Avulsion		35	13.6	14	20	11	3	6	7	8	1
			100	27	81	52	23	16	30	52†	4†
*DHT injuries*	152										
Enamel (infraction)		4	2.6						0		
Enamel (fracture)		35	23.0	1			1		2		
Enamel–dentin		67	44.1		12	9	1		4	2	4
Enamel–dentin–pulp		14	9.2	4	8	7	2		5		
Crown–root		27	17.8	13					3	3	6
Root		5	3.3	2					0	2	
			100			16				7‡	10‡
*PDL + DHT injuries*	159										
				11	61	48	7	11		13§	9§
Abbreviations in this row:					E(I)	E(F)	ED	EDP	CR	R	Total
E(I) = Enamel (infraction)				Concussion	7	19	36	3	2	1	68
E(F) = Enamel (fracture)				Subluxation	3	3	23	3	1	0	33
ED = Enamel–dentin				Lateral luxation	4	7	16	4	6	3	40
EDP = Enamel–dentin–pulp				Extrusion	3	0	1	0	0	0	4
CR = Crown–root				Intrusion	0	0	5	0	1	0	6
R = Root				Avulsion	0	5	3	0	0	0	8
Total	569			Total:	17	34	84	10	10	4	159

*DHT*  dental hard tissue, *PDL*  periodontal ligament

†Concussion: apical osteolysis (*n* = 1); subluxation: pulp canal obliteration (*n* = 7), prosthetic treatment (*n* = 2); lateral luxation: pulp canal obliteration (*n* = 11), apical osteolysis (*n* = 5), inflammatory replacement resorption (*n* = 2); extrusion: pulp canal obliteration (*n* = 2), apical osteolysis (*n* = 1); intrusion: apical osteolysis (*n* = 2), inflammatory resorption (*n* = 2), surface resorption (*n* = 10), extraction (*n* = 1); avulsion: inflammatory resorption (*n* = 4), pulp canal obliteration (*n* = 2), surface resorption (*n* = 2), removal (*n* = 7), prosthetic treatment (*n* = 1)

‡Enamel–dentin: pulp canal obliteration (*n* = 2), conservative treatment (*n* = 3), apicoectomy (*n* = 1); crown–root: surface resorption (*n* = 2), apical osteolysis (*n* = 1), extraction (*n* = 5), prosthetic treatment (*n* = 1); root: pulp canal obliteration (*n* = 2). §Apical osteolysis (*n* = 4), inflammatory (*n* = 2) or surface (*n* = 3) resorption, pulp canal obliteration (*n* = 4), apicoectomy (*n* = 1), extraction (*n* = 7), prosthetic treatment (*n* = 1)

While 536 previously injured teeth (89.6%) did not require treatment, an implant had been placed in 12 (2.0%) of the tooth sites and was being planned in another 15 (2.5%). A further 23 (3.8%) sites were restored by different means and 12 (2.0%) were left edentulous. A Pearson’s Chi-squared test revealed a strong, but not significant, tendency for implant treatment following TDI (*p* = 0.07) (Table 9).

**Table 9:** Cross-sectional subsample of patients: indications for implant treatment after previous injury to any affected teeth

	Restoration			Indication for implant treatment						Non-implant measures taken								
Types of traumatic injury	Not indicated			Implant placed			Implant planned			Restored			Left edentulous			All tooth sites		
(PDL, DHT, PDL + DHT)	*n*	%	%	*n*	%	%	*n*	%	%	*n*	%	%	*n*	%	%	*n*	%	%
PDL injuries	259		90.6	5		1.7	6		2.1	6		2.1	10		3.5	286		100
% based on injury types		48.3			41.7			40.0			26.1			83.3			47.8	
DHT injuries	132		86.3	7		4.6	3		2.0	9		5.9	2		1.3	153		100
% based on injury types		24.6			58.3			20.0			39.1			16.7			25.6	
PDL + DHT injuries	145		91.2	0		0.0	6		3.8	8		5.0	0		0.0	159		100
% based on injury types		27.1			0.0			40.0			34.8			0.0			26.6	
All injuries	536		89.6	12		2.0	15		2.5	23		3.8	12		2.0	598		100
Total %		100			100			100			100			100			100	

*DHT*  dental hard tissue, *PDL* periodontal ligament

Likelihood of implant treatment after traumatic dental injuries: *p* = 0.007 (Pearson's Chi-squared test)

Avulsions, due to their severity and extensive treatment requirements, were evaluated in detail. The total retrospective sample had included 298 avulsed teeth (Table 4); 44 of these (42 permanent and 2 deciduous) teeth or tooth sites could be followed up, 34 of them having been reimplanted while 10 were not.

Seventeen of the 44 teeth (38.6%) had been successfully preserved, while 9 (20.5%) were not considered for replantation, 5 sites (11.4%) had been managed by orthodontic gap closure, 4 (9.1%) by transplanting deciduous canines, 4 (9.1%) by fixed prostheses and 3 (6.8%) by implant treatment, while the remaining 2 (4.5%) were previously avulsed deciduous teeth. The mean Periotest values obtained for the previously injured teeth were 4.91 ± 4.53 (− 3 to + 29) based on the PDL injuries, 6.55 ± 7.25 based on the DHT and 5.34 ± 5.82 based on the PDL + DHT injuries. A Kruskal–Wallis test revealed a certain tendency, short of statistical significance, for the Periotest values to be associated with the injury types (*p* = 0.087).

## Discussion

This study was based on a sample of several thousand TDIs (*n* = 4909) from 2758 patients. The distribution of these traumatic injuries was found to be similar to other studies [1, 3, 12]. Moreover, this retrospective and cross-sectional analysis of patients, presenting at a German center, first and foremost, revealed a considerable gender discrepancy. Males outnumbered female patients by a factor of 1.65 and this distribution held true even when based only on the deciduous teeth. Perhaps, unsurprisingly, TDIs are well known to vary with gender and age. With regard to gender distribution, Petti et al. reported in a meta-analysis a global prevalence ratio of 1.43 and also suggested a 34 to 52% higher likelihood for males to experience dental trauma [3]. In other studies, male predominance has been found to range from 1.5 to 2.5 times [13–17] due to their statistically greater involvement in contact sports, fighting, occupational hazards and car accidents [12, 17–20]. Eslamipour et al. reported the prevalence of dental trauma to the permanent incisors as being 24% in 9- to 14-year-old patients, where the prevalence in girls was 18.8% compared to the significantly higher rate of 29.9% in boys [2].

The present study shows a continuous age gradient, with the first decade of life predominating and a clear majority of all patients (66.1%) being under 20 years old when the accidents occurred. The injury types differed for the permanent vs. the deciduous teeth and, with regard to the likelihood of sequelae, this notably included the extraction of teeth. There was a strong tendency for TDIs to entail implant treatment, while follow-up examinations revealed an 89.8% rate of tooth survival and a 60.2% rate of sequelae.

Consistent with a Chilean study where luxation trauma accounted for 70.4% of injuries to the deciduous teeth [14], in the present study the PDLs were twelve times more numerous than the DHT injuries to this dentition (Table 3). It has been noted that minor periodontal injuries may be underreported by going clinically unnoticed or due to parents not seeking a dentist in the absence of distinct symptoms or bleeding [21–25]. In a Turkish study, periodontal injuries were shown to account for 84.7% of injuries to the deciduous teeth, regardless of age or gender [26]. In the present study, 18.9% of the injured deciduous teeth were removed due to periodontal injuries. A series of retrospective cohort studies (follow-up ≥ 1 year) identified pulp necrosis, pulp canal obliteration, premature tooth loss and root resorption as the main sequelae of deciduous tooth trauma within 1 year [27–29].

Unlike the injury types (PDL versus DHT), the injury subtypes did not differ very much among the permanent and deciduous teeth. Notable exceptions included concussions (35.4% vs. 14.5% of PDL injuries) and fractures confined to the enamel (22.1% vs. 41.8% of DHT injuries) as opposed to enamel–dentin injuries (49.1% vs. 20.0%). PDL injuries to the permanent teeth were mainly found to include concussion (35.4%), lateral luxation (27.4%) and subluxation (22.0%). Cases of avulsion accounted for 7.4%. Regarding all injuries (to both permanent and deciduous teeth), trauma to the enamel or enamel–dentin fractures without pulp involvement accounted for 60% (22.6% plus 48.4%, respectively) of the DHT injuries. Hence, the latter (48.4%) were by far the most frequent subtype of hard-tissue injuries overall. Reviews from around the world (Nigeria, India, Canada and Chile) concluded that dental trauma mainly occurred to the enamel (63.7 to 80%), followed by enamel–dentin fractures (15.9 to 17.2%) or as uncomplicated crown fractures (32.9%) and subluxation (31.7%) [14, 30–32], whereas in a Brazilian study of all age groups, periodontal injuries were identified as the main type of dentoalveolar trauma [15, 33].

As the major findings of the present study concern sequelae, it is useful to provide a brief discussion of the mechanisms. Notable examples of complications following dental trauma would be pulp necrosis, apical periodontitis, clinical crown discoloration, fistula formation or inflammatory resorption. DHT injuries may facilitate bacterial colonization, inflammation and necrosis of the pulp [34–37]. Pulp survival has been reported to be 95 to 98% for uncomplicated crown fractures but only 63 to 94% for complicated crown fractures, however, after timely and correct treatment, long-term vitality may realistically be expected [37, 38]. After root fractures, pulp survival has been found in 60 to 80% of cases [39–42] and necrosis to be closely associated with the severity of the neurovascular supply disruption [43]. Therefore, while pulp necrosis is an unlikely scenario following isolated crown fractures if properly treated [44, 45], combined injuries (e.g., crown fracture plus subluxation) would weaken the pulp defense [33, 44] and increase the risk of necrosis by affecting not only the apical neurovascular bundle but also the periodontal fibers [44–46]. PDL injuries, which accounted for the majority of cases in the present study, may cause various forms of root resorption. Pulp necrosis is significantly more likely to occur in dislocated teeth with fully developed roots [47–49] and has been reported, depending on the severity of the trauma, to affect 17 to 100% of dislocated teeth [48, 50]. PDL injuries of the lateral-luxation, avulsion or intrusion type will often entail more serious complications such as external or replacement resorption, with lateral luxation resulting in soft-tissue damage and fracture of the vestibular bone lamella. Within the cross-sectional subsample reported here, only 175 of the 569 previously injured permanent teeth (30.8%) neither had sequelae nor required treatment. Conversely, 58 injured teeth (10.2%) were lost by the time of the follow-up examination; almost half of these losses (48.3%) occurred following PDL injuries. Informed on-site behavior and making the correct initial treatment decisions are essential to a favorable prognosis of traumatically injured teeth, which, as has been pointed out previously [43], will always depend on the type of trauma sustained, the length of time from the point of the accident to the emergency treatment, and the quality of the treatment.

The treatment of a TDI may be considered successful once healing of the pulp and periodontal soft tissue has been accomplished and the tooth is asymptomatic, exhibiting vitality, and appropriately positioned. In addition, the tooth should exhibit normal clinical and radiographic characteristics including an intact height of the alveolar bone as well as properly sealed root structures, with the root growth either completed or continuing. It is also a fact, however, that dental trauma can always entail sequelae which may vary in nature and severity; these often do not become manifest until months or, indeed, years after the event. Hence, early detection is the key to preventing long-term consequences; this can only be attained by conscientiously implementing and motivating patients to attend periodic recall visits in order to meticulously conduct all the required follow-up examinations.

### Limitations

Valuable information on the prognosis of TDIs was collected during the analysis. However, certain limitations were present due to the study design. Compared to retrospective studies, prospective studies can often collect more profound data. However, collecting the necessary data in the context of the initial treatment is questionable from an ethical point of view and difficult to integrate into the treatment process.

The data evaluated were taken from patient records, trauma documentation forms, radiographs and photographs. The evaluated TDIs were primarily treated by 23 different dentists or oral surgeons. Some of these practitioners were at the beginning of their professional careers, while others had several years of experience. Consequently, the prognosis of the affected teeth would be related to the clinical experience and competence of the individual dentist as it was they who had made the primary therapeutic decision.

The different treatment concepts applied, which have changed over the years due to current recommendations, may mean that there is a limit to the validity of the results. The TDIs that occurred during the period of 8 years were reexamined. The longer, historically, that the TDI had occurred, the less likely the patient would present for a recall examination in this study. Some patients presented regularly for a follow-up examination so that the necessary treatments could be performed early. Other follow-up patients presented for the first time after their primary care or had intermediate checkups and treatments performed by their dentists.

## Conclusion

Based on the findings of the present analysis, it can be concluded that males were more frequently affected by TDIs than females. Most patients had suffered from TDI before they had turned 10 years of age. Overall, the enamel–dentin fracture was found to be the most frequent injury, followed by concussions and lateral luxations.

## Data Availability

The data that support the findings of this study are available on request from the corresponding author. The data are not publicly available due to privacy or ethical restrictions.

## Abbreviations

DHT: Dental hard tissue
PDL: Periodontal ligament
TDIs: Traumatic dental injuries

## References

[1] Glendor U (2008). Epidemiology of traumatic dental injuries—a 12 year review of the literature. Dent Traumatol.

[2] Eslamipour F, Iranmanesh P, Borzabadi-Farahani A (2016). Cross-sectional study of dental trauma and associated factors among 9- to 14-year-old school children in Isfahan, Iran. Oral Health Prev Dent.

[3] Petti S, Glendor U, Andersson L (2018). World traumatic dental injury prevalence and incidence, a meta-analysis—one billion living people have had traumatic dental injuries. Dent Traumatol.

[4] Andersson L (2013). Epidemiology of traumatic dental injuries. Pediatr Dent.

[5] Richards D (2018). One billion people have experienced a traumatic dental injury. Evid Based Dent.

[6] Fasciglione D, Persic R, Pohl Y, Filippi A (2007). Dental injuries in inline skating—level of information and prevention. Dent Traumatol.

[7] Lahti H, Sane J, Ylipaavalniemi P (2002). Dental injuries in ice hockey games and training. Med Sci Sports Exerc.

[8] Lee JY, Divaris K (2009). Hidden consequences of dental trauma: the social and psychological effects. Pediatr Dent.

[9] Cortes MI, Marcenes W, Sheiham A (2002). Impact of traumatic injuries to the permanent teeth on the oral health-related quality of life in 12–14-year-old children. Community Dent Oral Epidemiol.

[10] Spinas E, Melis A, Savasta A (2006). Therapeutic approach to intrusive luxation injuries in primary dentition. A clinical follow-up study. Eur J Paediatr Dent.

[11] Krastl G, Filippi A, Weiger R (2020). Initial management of dental trauma: musts, shoulds, and cans. Quintessence Int.

[12] Azami-Aghdash S, Ebadifard Azar F, Pournaghi Azar F, Rezapour A, Moradi-Joo M, Moosavi A, GhertasiOskouei S (2015). Prevalence, etiology, and types of dental trauma in children and adolescents: systematic review and meta-analysis. Med J Islam Repub Iran.

[13] Lam R, Abbott P, Lloyd C, Lloyd C, Kruger E, Tennant M (2008). Dental trauma in an Australian rural centre. Dent Traumatol.

[14] Díaz JA, Bustos L, Brandt AC, Fernández BE (2010). Dental injuries among children and adolescents aged 1–15 years attending to public hospital in Temuco, Chile. Dent Traumatol.

[15] Navabazam A, Farahani SS (2010). Prevalence of traumatic injuries to maxillary permanent teeth in 9- to 14-year-old school children in Yazd, Iran. Dent Traumatol.

[16] Noori AJ, Al-Obaidi WA (2009). Traumatic dental injuries among primary school children in Sulaimani city, Iraq. Dent Traumatol.

[17] Naidoo S, Sheiham A, Tsakos G (2009). Traumatic dental injuries of permanent incisors in 11- to 13-year-old South African schoolchildren. Dent Traumatol.

[18] Eyuboglu O, Yilmaz Y, Zehir C, Sahin H (2009). A 6-year investigation into types of dental trauma treated in a paediatric dentistry clinic in Eastern Anatolia region. Turkey Dent Traumatol.

[19] Altun C, Ozen B, Esenlik E, Guven G, Gürbüz T, Acikel C, Basak F, Akbulut E (2009). Traumatic injuries to permanent teeth in Turkish children, Ankara. Dent Traumatol.

[20] David J, Astrøm AN, Wang NJ (2009). Factors associated with traumatic dental injuries among 12-year-old schoolchildren in South India. Dent Traumatol.

[21] Andreasen JO, Andreasen FM, Skeie A, Hjørting-Hansen E, Schwartz O (2002). Effect of treatment delay upon pulp and periodontal healing of traumatic dental injuries—a review article. Dent Traumatol.

[22] Saroglu I, Sönmez H (2002). The prevalence of traumatic injuries treated in the pedodontic clinic of Ankara University, Turkey, during 18 months. Dent Traumatol.

[23] Wilson CF (1995). Management of trauma to primary and developing teeth. Dent Clin N Am.

[24] Cunha RF, Pugliesi DM, de Mello Vieira AE (2001). Oral trauma in Brazilian patients aged 0–3 years. Dent Traumatol.

[25] Kramer PF, Zembruski C, Ferreira SH, Feldens CA (2003). Traumatic dental injuries in Brazilian preschool children. Dent Traumatol.

[26] Avsar A, Topaloglu B (2009). Traumatic tooth injuries to primary teeth of children aged 0–3 years. Dent Traumatol.

[27] Lauridsen E, Blanche P, Amaloo C, Andreasen JO (2017). The risk of healing complications in primary teeth with concussion or subluxation injury—a retrospective cohort study. Dent Traumatol.

[28] Lauridsen E, Blanche P, Yousaf N, Andreasen JO (2017). The risk of healing complications in primary teeth with extrusive or lateral luxation—a retrospective cohort study. Dent Traumatol.

[29] Lauridsen E, Blanche P, Yousaf N, Andreasen JO (2017). The risk of healing complications in primary teeth with intrusive luxation: a retrospective cohort study. Dent Traumatol.

[30] Taiwo OO, Jalo HP (2011). Dental injuries in 12-year old Nigerian students. Dent Traumatol.

[31] Gupta S, Kumar-Jindal S, Bansal M, Singla A (2011). Prevalence of traumatic dental injuries and role of incisal overjet and inadequate lip coverage as risk factors among 4–15 years old government school children in Baddi-Barotiwala Area, Himachal Pradesh, India. Med Oral Patol Oral Cir Bucal.

[32] Fakhruddin KS, Lawrence HP, Kenny DJ, Locker D (2008). Etiology and environment of dental injuries in 12- to 14-year-old Ontario schoolchildren. Dent Traumatol.

[33] Gulinelli JL, Saito CT, Garcia-Júnior IR, Panzarini SR, Poi WR, Sonoda CK, Jardim EC, Faverani LP (2008). Occurrence of tooth injuries in patients treated in hospital environment in the region of Araçatuba, Brazil during a 6-year period. Dent Traumatol.

[34] Mjör IA (2001). Pulp-dentin biology in restorative dentistry. Part 5: clinical management and tissue changes associated with wear and trauma. Quintessence Int.

[35] Mjör IA (2002). Pulp-dentin biology in restorative dentistry. Part 7: the exposed pulp. Quintessence Int.

[36] Love RM, Jenkinson HF (2002). Invasion of dentinal tubules by oral bacteria. Crit Rev Oral Biol Med.

[37] Lauridsen E, Hermann NV, Gerds TA, Ahrensburg SS, Kreiborg S, Andreasen JO (2012). Combination injuries 1. The risk of pulp necrosis in permanent teeth with concussion injuries and concomitant crown fractures. Dent Traumatol.

[38] de Blanco LP (1996). Treatment of crown fractures with pulp exposure. Oral Surg Oral Med Oral Pathol Oral Radiol Endod.

[39] Cvek M, Andreasen JO, Borum MK (2001). Healing of 208 intra-alveolar root fractures in patients aged 7–17 years. Dent Traumatol.

[40] Andreasen JO, Andreasen FM, Mejàre I, Cvek M (2004). Healing of 400 intra-alveolar root fractures. 1. Effect of pre-injury and injury factors such as sex, age, stage of root development, fracture type, location of fracture and severity of dislocation. Dent Traumatol.

[41] Andreasen JO, Andreasen FM, Mejàre I, Cvek M (2004). Healing of 400 intra-alveolar root fractures. 2. Effect of treatment factors such as treatment delay, repositioning, splinting type and period and antibiotics. Dent Traumatol.

[42] Cvek M, Mejàre I, Andreasen JO (2002). Healing and prognosis of teeth with intraalveolar fractures involving the cervical part of the root. Dent Traumatol.

[43] Zaleckiene V, Peciuliene V, Brukiene V, Drukteinis S (2014). Traumatic dental injuries: etiology, prevalence and possible outcomes. Stomatologija.

[44] Robertson A (1998). A retrospective evaluation of patients with uncomplicated crown fractures and luxation injuries. Endod Dent Traumatol.

[45] Robertson A, Andreasen FM, Andreasen JO, Norén JG (2000). Long-term prognosis of crown-fractured permanent incisors. The effect of stage of root development and associated luxation injury. Int J Paediatr Dent.

[46] Viduskalne I, Care R (2010). Analysis of the crown fractures and factors affecting pulp survival due to dental trauma. Stomatologija.

[47] Lee R, Barrett EJ, Kenny DJ (2003). Clinical outcomes for permanent incisor luxations in a pediatric population. II. Extrusions. Dent Traumatol.

[48] Nikoui M, Kenny DJ, Barrett EJ (2003). Clinical outcomes for permanent incisor luxations in a pediatric population. III. Lateral luxations. Dent Traumatol.

[49] Ferrazzini Pozzi EC, von Arx T (2008). Pulp and periodontal healing of laterally luxated permanent teeth: results after 4 years. Dent Traumatol.

[50] Humphrey JM, Kenny DJ, Barrett EJ (2003). Clinical outcomes for permanent incisor luxations in a pediatric population. I. Intrusions. Dent Traumatol.

